# Identifying small groups of foods that can predict achievement of key dietary recommendations: data mining of the UK National Diet and Nutrition Survey, 2008–12

**DOI:** 10.1017/S1368980016000185

**Published:** 2016-02-16

**Authors:** Philippe J Giabbanelli, Jean Adams

**Affiliations:** UKCRC Centre for Diet and Activity Research (CEDAR), MRC Epidemiology Unit, University of Cambridge School of Clinical Medicine, Institute of Metabolic Science, Cambridge CB2 0QQ, UK

**Keywords:** Data mining, Diet, Dietary assessment, Dietary pattern analysis, Nutrition

## Abstract

**Objective:**

Many dietary assessment methods attempt to estimate total food and nutrient intake. If the intention is simply to determine whether participants achieve dietary recommendations, this leads to much redundant data. We used data mining techniques to explore the number of foods that intake information was required on to accurately predict achievement, or not, of key dietary recommendations.

**Design:**

We built decision trees for achievement of recommendations for fruit and vegetables, sodium, fat, saturated fat and free sugars using data from a national dietary surveillance data set. Decision trees describe complex relationships between potential predictor variables (age, sex and all foods listed in the database) and outcome variables (achievement of each of the recommendations).

**Setting:**

UK National Diet and Nutrition Survey (NDNS, 2008–12).

**Subjects:**

The analysis included 4156 individuals.

**Results:**

Information on consumption of 113 out of 3911 (3 %) foods, plus age and sex was required to accurately categorize individuals according to all five recommendations. The best trade-off between decision tree accuracy and number of foods included occurred at between eleven (for fruit and vegetables) and thirty-two (for fat, plus age) foods, achieving an accuracy of 72 % (for fat) to 83 % (for fruit and vegetables), with similar values for sensitivity and specificity.

**Conclusions:**

Using information on intake of 113 foods, it is possible to predict with 72–83 % accuracy whether individuals achieve key dietary recommendations. Substantial further research is required to make use of these findings for dietary assessment.

The intention of many dietary assessment methods is to capture information on all foods consumed, or at least those believed to make the largest contribution to total intake^(^ ^1^ ^)^, in order to estimate total nutrient intake. For some purposes, this detailed estimation of total nutrient intake may lead to collection of much redundant data. This is particularly the case when assessing adherence with policy targets and messages such as ‘five-a-day’ portions of fruit and vegetables.

The collection of substantial redundant information places unnecessary burden on research participants and unnecessarily uses scarce research resources. To take a first step to overcoming this problem, we applied data mining techniques to explore how many, and which, foods intake information was required on to accurately predict achievement, or not, of key dietary recommendations.

## 

### Data mining, an overview

Unlike traditional statistical approaches such as multiple regression, data mining allows multiple non-linear relationships and interaction effects to be efficiently captured^(^ ^2^ ^,^ ^3^ ^)^. Several data mining tools exist. In the present study we used ‘classifiers’. A classifier is a function that labels individuals on an outcome (e.g. achieving a dietary recommendation or not) based on a group of predictor variables (e.g. how much of each individual food was consumed). The analysis package is first provided with a ‘training set’ of individual-level data in which both the outcome and the predictor variables are known, and uses this to learn how the predictor variables are related to the outcome. This produces the classifier function, which can then be used to infer the outcome in a new case based on just the predictor variables. Finally, the accuracy of the classifier is evaluated on a new ‘testing set’ of data.

There are numerous ways to build classifiers. We used ‘decision trees’^(^ ^2^ ^,^ ^4^ ^,^ ^5^ ^)^. Decision trees provide a graphical illustration of a classifier composed of a number of predictor variables. A decision tree involves repeated ‘cuts’ of the data according to the level of included predictor variables to identify groups of individuals who are similar in terms of the outcome variable of interest. This produces a decision tree where the path from the root to the outcome corresponds to successive ‘cuts’, or divisions, of the population.

Figure 1 provides a simplified, hypothetical example of a decision tree where the intention is to identify whether or not individuals achieve the recommended intake of fruit and vegetables (the outcome) using information on the consumption of carrots and white bread (the two predictor variables). Figure 1(a) shows the decision tree based on the ‘cuts’ represented in Fig. 1(b), the latter being a simple graphical plot of consumption of both carrots and white bread with all individuals labelled according to whether or not they achieve the recommended intake of fruit and vegetables. In terms of meeting fruit and vegetable recommendations there appear to be five ‘clusters’ of participants in Fig. 1(b). A series of ‘cuts’ can isolate these clusters. The first cut (labelled ‘A’ in Fig. 1(a) and 1(b)) divides the population according to consumption of carrots. The next two cuts (labelled ‘B’ and ‘C’) then divide the resulting two groups according to consumption of white bread. Finally, a fourth cut (labelled ‘D’) divides those with a medium carrot and medium white bread intake according to a more fine-grained assessment of carrot intake.

**Fig. 1:**
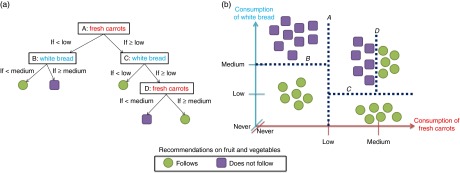
(colour online) Schematic illustration of a decision tree (a) and how this is formed through repeated ‘cuts’ of the data (b)

To build decision trees with different numbers of predictor variables, the minimum number of individual cases that can be further divided by a subsequent ‘cut’ is varied. If a small group of individuals can be further subdivided, a sizeable tree including many predictor variables can result. However, if limits are placed on the minimum size of group that can be further subdivided, a smaller decision tree, including fewer predictor variables, results. In the current study we made use of this feature to explore the effect of including more or fewer predictor variables on the accuracy of decision trees.

A small number of studies have applied data mining techniques to nutritional data. These have focused primarily on dietary pattern analysis, exploring which dietary components are predictive of a range of health outcomes^(^ ^6^ ^–^ ^9^ ^)^. However, we are not aware of any other uses of data mining to identify which foods are predictive of achievement, or not, of key dietary recommendations.

### Aim

Our aim was to use data mining techniques to determine the number of foods that intake information was required on to accurately predict achievement, or not, of dietary recommendations for intake of fruit and vegetables, free sugars, sodium, fat and saturated fat.

## Methods

We built decision trees for achievement of key dietary recommendations using data from the first four years of the rolling programme of the UK’s national dietary surveillance data set: the National Diet and Nutrition Survey (NDNS).

### Data source

The NDNS is an annual cross-sectional survey assessing the diet, nutrient intake and nutritional status of the general population aged 18 months and upwards living in private households in the UK^(^ ^10^ ^)^. Since 2008 an annual ‘rolling programme’ has been in place, allowing data to be combined over years. We used data from years 1–4 of this programme, collected in 2008–12.

The NDNS aims to collect data from a sample of 1000 respondents per year: at least 500 adults (aged 19 years and older) and at least 500 children (aged 1·5 to 18 years). Households across the UK are selected to take part in the NDNS using a multistage probability design. In each wave, a random sample of primary sampling units is selected for inclusion. These are small geographical areas that allow more efficient data collection by enabling it to be geographically focused. Within these primary sampling units, private addresses are randomly selected for inclusion. If, on visiting, it is found that more than one household lives at a particular address, one is randomly selected for inclusion. Within participating households, up to one adult and one child are randomly selected to take part as ‘respondents’. Data collection includes completion of a 4 d estimated food diary, where participants estimate the weight of foods consumed using food labels and household measures^(^ ^11^ ^)^.

NDNS data were obtained from the UK Data Archive, an online resource that makes research data available to the UK research community.

### Inclusion and exclusion criteria

NDNS participants were included in the analysis if they completed 3 or 4 d of the estimated food diary. As recommendations for fruit and vegetable intake apply only to those aged 11 years or older, children aged less than 11 years were excluded from this component of the analysis.

### Outcomes of interest: achievement of dietary recommendations

Information on which foods were consumed, and how much participants estimated was consumed, was combined with nutritional information to determine mean daily intake of fruit and vegetables (80 g portions) and sodium (milligrams), as well as mean daily percentage of energy derived from fat, saturated fat and free sugars, for each individual. This information was then used to determine whether or not each individual met international, or UK, recommendations for these variables.

We used UK recommendations for fruit and vegetable and sodium intakes, as these have been graded according to age. It is recommended that individuals aged 11 years and older consume at least five 80 g portions of fruit and vegetables daily. This includes a maximum of one portion of juice, with additional juice portions not counted. For sodium, current UK recommendations are that those aged 11 years and older consume no more than 2400 mg/d; children aged 7–10 years, no more than 2000 mg/d; children aged 4–6 years, no more than 1200 mg/d; and children aged 1–3 years, no more than 800 mg/d^(^ ^12^ ^)^.

The WHO recommends population food and nutrient intake goals for the avoidance of diet-related diseases. These state that no more than 30 % of energy should be derived from fat, no more than 10 % from saturated fat and no more than 10 % from free sugars^(^ ^13^ ^)^.

### Predictor variables of interest: foods consumed

In total, 3911 different foods (including drinks) have been recorded in NDNS food diaries. We used total estimated weight (in grams) of each individual food eaten by each individual as potential predictor variables. Age and sex were also included as potential predictor variables. The use of including markers of socio-economic position (education, income and social class) as potential predictor variables was explored, but these were found to add no additional increase in accuracy over and above age, sex and individual foods. Decision trees reported here do not include any socio-economic predictor variables.

### Data analysis

Our analysis scripts and detailed decision trees are available at https://osf.io/znv82. In all cases except sodium, the proportion of individuals achieving the recommendations was substantially less than 50 %; for sodium substantially more than 50 % of individuals achieved the recommendations (Table 2). As detailed in the online supplementary material, this imbalance in outcome variables can lead to low-quality classifiers. To correct this, we pre-processed the data using the Synthetic Minority Over-sampling TEchnique (SMOTE)^(^ ^14^ ^)^, which creates new cases for the group that accounted for less than 50 % of participants by interpolating between existing cases that lie together. WEKA software^(^ ^15^ ^)^ was then used to build decision trees using the J48 algorithm and error pruning.

**Table 1:** Comparison of the analytical sample with the UK population

	Adults aged 19 years or older				Children aged <19 years			
	Analytical sample (*n* 2083)		UK population		Analytical sample (*n* 2073)		UK population	
Variable	*n*	%	*n*	%	*n*	%	*n*	%
Female	1182	56·8	25 198 773	51·5	1007	48·6	6 955 262	48·8
Age (adults)								
19–29 years	296	14·2	9 447 071	19·3	–		–	
30–39 years	390	18·7	8 319 926	17·0	–		–	
40–49 years	425	20·4	9 268 735	18·9	–		–	
50–59 years	363	17·4	7 708 532	15·8	–		–	
60–64 years	181	8·7	3 807 975	7·8	–		–	
≥65 years	428	20·6	10 377 127	21·2	–		–	
Age (children)								
0–4 years	–		–		499	24·1	3 913 953	27·5
5–9 years	–		–		583	26·4	3 516 615	24·7
10–14 years	–		–		547	26·4	3 669 326	25·7
15–18 years	–		–		444	21·4	3 152 919	22·1

For each outcome of interest we built a series of decision trees with different numbers of predictor variables by varying the minimum number of individual cases that could be further divided. For each of the decision trees built, we calculated the number of predictor variables used and overall accuracy in correctly classifying individuals. We used the standard tenfold cross-validation procedure^(^ ^16^ ^)^ in which the entire eligible NDNS data set was split into ten approximately equally sized parts. Nine parts were used in turn as training sets and the remaining tenth part was used as the testing set. The ability of decision trees to correctly identify those who achieved the recommendations (sensitivity) and those who did not (specificity) was also calculated. Adaptive sampling was used to identify the maximum overall accuracy that could be achieved, as well as the optimum trade-off between minimizing the number of predictor variables and maximizing the overall accuracy.

## Results

Overall, 91 % of households eligible for inclusion agreed to take part in the first four waves of NDNS. Within these, 56 % (2083 adults and 2073 children; 4156 participants in total) of individuals selected to take part completed 3 or 4 d of the estimated food diary and were included in the analysis for sodium, free sugars, fat and saturated fat. Of these 4156 participants, 2967 (71·4 %) were aged 11 years or older and included in the analysis for fruit and vegetables. There were no missing data on sex or age.

The distributions of age and sex in the analytical sample compared with the UK population as a whole are shown in Table 1. As the NDNS sample contains relatively equal numbers of children aged 18 years or younger and adults, distributions are provided separately for adults and children in Table 1. The main differences in the age and sex distributions between the analytical sample and the UK population were that the analytical sample had a higher proportion of adult women and a lower proportion of young adults (aged 19–29 years) than the UK population.

**Fig. 2:**
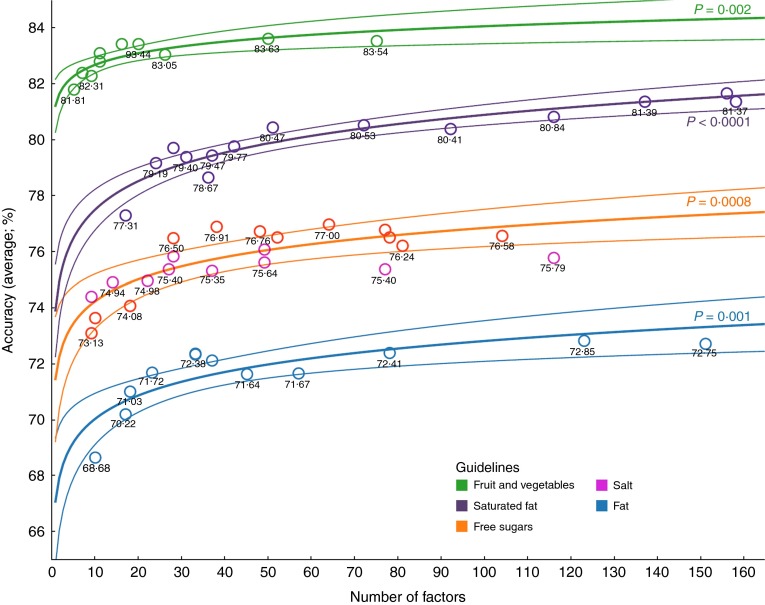
(colour online) Overall accuracy (with 95 % confidence margins) of decision trees *v*. the number of predictor variables included, using data mining techniques on the nutritional intake of 4156 individuals (2967 individuals for fruit and vegetables) from the UK National Diet and Nutrition Survey (2008–12)

Figure 2 shows the overall accuracy of decision trees for each of the five outcomes plotted against the number of predictor variables in decision trees. Overall accuracy ranged from 69 % (fat; ten predictor variables) to 84 % (fruit and vegetables; fifty predictor variables) depending on the outcome of interest and number of predictor variables included. For all guidelines but sodium, the relationship between the number of predictor variables and the accuracy was best described using a logarithmic trend model (*P*<0·01 in all cases). Thus, increasing the number of predictor variables from about ten to thirty improved the accuracy by a maximum of about five percentage points, but beyond this adding even a large number of additional predictor variables yielded only a very small additional improvement. We were unable to fit any function to the relationship between accuracy and number of predictor variables for sodium.

Table 2 provides information on the decision tree for each outcome that represented the best trade-off between accuracy and number of predictor variables. Information on the most accurate possible tree for each outcome is also shown in Table 2. Between eleven (for fruit and vegetables) and thirty-three (for fat) predictor variables provided the best trade-off to identify whether individuals achieved each of the recommendations, achieving overall accuracy of 72 % (for fat) to 83 % (for fruit and vegetables). Adding further predictor variables beyond this improved accuracy by a maximum of 2 % (for saturated fat) and less than 1 % (for all other outcomes). Sensitivity and specificity were similar to overall accuracy for fruit and vegetables and free sugars (and for saturated fat when the maximum number of predictor variables was included). However, specificity was higher than sensitivity for fat (and saturated fat), but the reverse was seen for sodium. Predictor variables in decision trees with the best trade-off between accuracy and number of predictor variables accounted for between 13 % (for fat) and 31 % (for free sugars) of total intake of relevant outcome variables.

**Table 2:** Prevalence of achieving and not achieving dietary recommendations and accuracy of decision trees to predict this, using data mining techniques on the nutritional intake of 4156 individuals (2967 individuals for fruit and vegetables) from the UK National Diet and Nutrition Survey (2008–12)

	Fruit & vegetables	Free sugars	Sodium	Fat	Saturated fat
No. achieving recommendation without oversampling	656	1472	2524	1045	795
%	22·1	35·4	60·7	25·1	19·1
SMOTE oversampling %*	252 % (yes)	85 % (yes)	54 % (no)	197 % (yes)	322 % (yes)
No. achieving recommendation after oversampling	2309*	2679	2524	3103	3354
No. not achieving recommendation after oversampling	2311*	2684	2513	3111	3361
Decision tree with the best trade-off between accuracy and number of predictor variables					
Overall accuracy (%)	83·1	76·5	75·9	72·4	79·7
Sensitivity (%)	82·5	76·1	81·9	66·3	75·8
Specificity (%)	83·8	76·9	69·8	78·4	83·6
No. of predictor variables	11	28	28	33	28
% of all relevant food/nutrient (g) accounted for by predictor variables	21·0†	31·2	13·4	13·0	27·4
Most accurate decision tree					
Overall accuracy (%)	83·6	77·0	76·1	72·9	81·7
Sensitivity (%)	83·9	75·7	80·7	69·3	81·4
Specificity (%)	83·3	78·3	71·5	76·4	81·9
No. of predictor variables	50	64	49	123	156
% of all relevant food/nutrient (g) accounted for by predictor variables	30·8†	38·6	25·4	29·5	42·7

SMOTE, Synthetic Minority Over-sampling TEchnique.

* After oversampling using the SMOTE method (see online supplementary material).

† Percentage of all fruit and vegetables (g) recorded, not just those contributing to 5-a-day portions (specifically, fruit juice can contribute a maximum of only one 5-a-day portion).

Predictor variables used in decision trees with the best trade-off between accuracy and number of predictor variables are shown in Table 3. In total, 113 foods (3 % out of a total 3911 recorded as consumed), age and sex were included in the decision trees for all five outcomes. Overall, there was little overlap in predictor variables across outcomes. Age and two foods were included as predictor variables in the decision trees for three outcomes. A further six foods were included as predictor variables in the decision trees for two outcomes. The remaining 104 foods were included as predictor variables in only one decision tree.

**Table 3:** Predictor variables (individual foods, age and sex) included in decision trees for predicting achievement of five dietary recommendations, using data mining techniques on the nutritional intake of 4156 individuals (2967 individuals for fruit and vegetables) from the UK National Diet and Nutrition Survey (2008–12)

Dietary recommendation outcome					
Fat	Free sugars	Fruit & vegetables	Sodium	Saturated fat	Food name
Yes			Yes	Yes	Age
Yes					Alcoholic soft drinks, spirit based
		Yes			Almonds, kernel only: ground almonds
	Yes				Apple juice, unsweetened, cartons, pasteurized
	Yes				Apple juice, unsweetened, UHT
		Yes			Apples, eating, raw, flesh & skin only
Yes					Avocado pear, flesh only
			Yes		Bacon rashers, back, grilled, lean and fat
			Yes		Bacon rashers, back, not smoked, grilled, extra trim
			Yes		Baked beans in tomato sauce with pork sausages
Yes		Yes			Bananas, raw, flesh only
				Yes	Beefburger and onion, grilled
Yes					Black pudding, fried
	Yes				Blackcurrant juice drink, ready to drink, not low calorie
	Yes				Boiled sweets, barley sugar, butterscotch, glacier mints, hard candy
			Yes		Bread, white, crusty
			Yes	Yes	Bread, white, toasted
Yes					Bread, 50 % white and 50 % wholemeal flours
			Yes		Bread, white sliced, not fortified
			Yes		Brown sauce, bottled
			Yes		Brussels sprouts, fresh, boiled
Yes					Butter beans, dried, boiled
Yes				Yes	Butter, salted
				Yes	Butter, unsalted
	Yes				Carbonated beverages, no juice, not low calorie, canned
Yes	Yes			Yes	Carbonated beverages, no juice, not low calorie, not canned
		Yes			Celery, fresh, raw
Yes					Chapatti, brown, no fat
Yes				Yes	Cheese, cheddar, any other or for recipes
				Yes	Cheese, cheddar, English
			Yes		Cheese, soft full fat, Philadelphia type
Yes					Chicken fried in olive oil
				Yes	Children’s fromage frais fruit with added vitamin D
				Yes	Chocolate brownie, no nuts, purchased
				Yes	Chocolate-covered caramels, Cadbury Caramel
	Yes				Chocolate Swiss roll with butter cream, purchased
	Yes				Cola cherry cola, canned, not low calorie
	Yes				Cola, not canned, not low calorie, not caffeine free
Yes					Coleslaw, purchased, not low calorie
Yes					Cookies and biscuits with chocolate
				Yes	Cornetto type ice cream, chocolate or nut based
	Yes				Cranberry fruit juice drink, e.g. Ocean Spray
				Yes	Cream, double
	Yes				Cream egg
				Yes	Croissants, plain, not filled
	Yes				Drinking chocolate, instant, dry weight
			Yes		Fat spread (62–72 % fat), not polyunsaturated
	Yes				Fruit gums, wine gums
	Yes				Fruit juice drink, carbonated, not low calorie, not canned
	Yes				Fruit juice drink with 5 % fruit juice, ready to drink
				Yes	Fully coated chocolate biscuits with biscuit filling
Yes					Garlic bread, lower fat
			Yes		Ham, unspecified, not smoked, not canned
			Yes		Hamburger, Big Mac, McDonalds
	Yes				High juice, ready to drink, not blackcurrant or low calorie
	Yes				Ice lollies
	Yes				Jaffa Cakes
				Yes	Kit Kat
Yes					Lager, not canned, e.g. Heineken
Yes					Lager, not canned, e.g. Skol
Yes					Lamb scrag and neck, stewed, lean only
	Yes				Lemonade, not low calorie, not canned
				Yes	Light spreadable butter (60 % fat)
	Yes				Lucozade sport isotonic drink, not carbonated
Yes				Yes	Mayonnaise (retail)
			Yes	Yes	Milk chocolate bar
	Yes				Milk shake, thick style, takeaway
Yes					Milk, skimmed, after boiling
				Yes	Milk, whole pasteurized, winter
				Yes	Milk, whole pasteurized, summer
Yes					Mushrooms fried in olive oil
			Yes		Naan bread, plain
		Yes			Oatcakes
	Yes				Olive oil
		Yes			Onions, boiled
	Yes				Orange juice, unsweetened, UHT
Yes					Oven ready chips
			Yes		Papadums/poppadoms, fried in vegetable ghee
Yes					Pasta noodles, boiled
			Yes		Pasta noodles, egg, boiled
Yes					Pasta spaghetti, boiled, white
			Yes		Peanut butter, crunchy, not wholenut
		Yes			Pears, eating, raw, flesh & skin only, no core
Yes					Pepperami
				Yes	Petit Filous fromage frais
			Yes		Potato cakes (scones), purchased
Yes					Potatoes, new, boiled, skins eaten
			Yes		Potatoes, old, baked, flesh & skin
				Yes	Potatoes, old, mashed & butter
			Yes		Prawns, boiled, flesh only
			Yes		Reduced fat spread (41–62 %), not polyunsaturated
			Yes		Ribena, original blackcurrant drink, concentrate
	Yes				Robinsons Fruit Shoot
			Yes		Rolls, white, crusty
Yes		Yes		Yes	Sausage roll, flaky pastry, purchased
Yes					Sausages, pork, grilled
			Yes		Sausages, premium pork, grilled
Yes					Scrambled eggs with skimmed milk and no fat
Yes					Semi-sweet biscuit
			Yes		Sex
	Yes				Soya alternative to milk, sweetened plain
		Yes			Spinach, fresh, raw
				Yes	Spreadable butter (75–80 % fat)
	Yes				Sugar, white
				Yes	Super Noodles, Batchelors, as served
				Yes	Swiss roll, individual, chocolate coated, purchased
		Yes			Tomatoes, raw
			Yes		Turkey slices, unsmoked, pre-pack or deli
	Yes				Water for concentrated soft drinks, not diet
Yes					White chocolate buttons, mice
Yes					Whole milk, after boiling
Yes					Wine white, dry, not canned
	Yes			Yes	Yoghurt twin pot with cereal/crumble
		Yes			Yoghurt, Greek style, cows, natural, whole milk
			Yes		Yorkshire pudding, frozen

UHT, ultra-heat treated.

## Discussion

### Summary of results

The present study represents the first work we are aware of using data mining techniques to explore the number of foods that information is required on to predict achievement of dietary recommendations. In total, information on consumption of 113 of 3911 foods (3 %), plus age and sex was required to accurately categorize individuals according to all five dietary recommendations (fruit and vegetables, free sugars, sodium, fat and saturated fat). The best trade-off between decision tree accuracy and number of foods included was achieved at between eleven (for fruit and vegetables) and thirty-two (for fat, plus age) foods. These decision trees had an overall accuracy of 72 % (for fat) to 83 % (for fruit and vegetables), with similar values for sensitivity and specificity. Few individual foods were present in the decision tree for more than one dietary recommendation, although age was present in three.

### Strengths and limitations of methods

We used data from a population-based sample, meaning that our findings are likely to be generalizable across the UK and to other countries with similar dietary profiles. However, diets vary internationally^(^ ^17^ ^)^ and our results may not be more widely generalizable. The analytical sample had a slightly higher proportion of adult women and a lower proportion of younger adults (aged 19–29 years) than the UK population as a whole.

The data used were collected using ‘estimated’ food diaries where portion sizes were estimated but not weighed. These are considered to be one of the more accurate methods of measuring dietary intake^(^ ^18^ ^)^, meaning that both the predictor and outcome variables are likely to be valid. However, even estimated food diaries have their limitations, particularly in terms of participant burden and under-reporting of energy intake^(^ ^19^ ^,^ ^20^ ^)^. Doubly labelled water has been used to estimate total energy expenditure in a sub-sample of NDNS participants and compared with reported energy intake from food diaries. This reveals that reported energy intake is 12–34 % lower than estimated total energy expenditure, depending on the age of participants^(^ ^11^ ^)^. This mismatch may be due to intentional or unintentional misreporting; participants changing their food intake in response to recording it; or a variety of other reasons. However, misreporting is unlikely to affect all foods and nutrients equally. For example, participants may be more likely to misreport confectionery than vegetable intake. For this reason, misreporting is not adjusted for in NDNS and we have not adjusted for misreporting here.

Data mining using decision trees is computationally and statistically efficient. For example, inclusion of all 3911 foods consumed by NDNS participants in regression models with achievement of dietary recommendations as outcomes would be computationally, and statistically, demanding and unlikely to produce satisfactory results. Decision trees also produce transparent, and intuitively understandable, outputs (ours are provided at https://osf.io/znv82)^(^ ^21^ ^)^.

Many of foods included in the analysis had very skewed distributions. Indeed, the vast majority of foods in the database (3618) were eaten by less than 150 people. Decision trees seek to maximize information gain at each step, rather than working with the distribution as a whole as in traditional regression analysis. If an item is very discriminatory and helps differentiate between those who do and do not meet a particular guideline then it will be included, even if it is consumed by only a small number of people. Conversely, if an item is eaten by almost everyone but is not discriminatory, then it would be unlikely to be included. There was no overall trend between the proportion of participants who ate a food and the chance that that food was included in a decision tree (data not shown).

We used adaptive sampling to identify decision trees that achieved the best trade-off between accuracy and number of predictor variables included. Thus, instead of systematically calculating the accuracy of all decision trees including all possible number of predictor variables, we focused on identifying the relationship between accuracy and number of predictor variables (logarithmic in most cases) and where the optimum trade-off between accuracy and number of predictor variables occurred (i.e. where the logarithmic curve flattened out). This means we cannot be absolutely sure that we have identified the decision trees with the best trade-off between accuracy and number of predictor variables in all cases. However, given the very small additional improvements in accuracy achieved by the most accurate *v*. best trade-off decision trees, we are certainly likely to have identified the near-best trade-off decision trees.

We used estimated dietary records as our ‘gold standard’ tool for determining whether or not individuals achieved recommendations. Further work will be required to compare the accuracy of our decision trees with other methods of estimating who achieves dietary recommendations, such as FFQ.

### Interpretation and implications of findings and areas for future work

Our findings indicate that information on only a small number of foods is required to determine whether individuals achieve five important dietary recommendations. If such binary outcomes are the key outcome of interest, then more detailed dietary assessment methods may inappropriately use scarce research resources and be unnecessarily burdensome to participants.

While our results suggest that information on only a limited number of foods needs to be captured when assessing whether guidelines are met, substantial further research will be needed before these findings could be applied in the form of a new dietary assessment instrument. First, it would be helpful to replicate our analyses in a different, but comparable, sample. We have not done this as we are not aware of a comparable UK population-representative sample in whom diet diaries have been collected. Our decision trees used information on exact intake of 113 foods over 3–4 d. Assessing exact intake of a small number of foods may be no less burdensome for participants than assessing estimated intake of all foods using a food diary. Future work could compare the accuracy of decision trees based on exact intake of 113 foods, approximate intake of these foods (e.g. using the ordinal categories often used in FFQ), and exact and approximate intakes of foods at the food group, rather than individual food, level. Acceptability to research participants and resource implications of collecting the data required in all cases should also be compared.

Our analysis focused on which foods can be used to predict whether or not individuals achieve dietary recommendations. But it is not necessarily the case that it is the foods included in the decision tress which cause people to achieve the recommendations or not. A maximum of only 31 % of the total intake of relevant nutrients or foods was accounted for by predictor variables in decision trees with the best trade-off between accuracy and number of predictor variables. Thus, decision trees did not particularly include foods that account for the majority of intake of nutrients and foods of interest – as might be expected in an FFQ. The complex relationships between individual foods included in our decision trees and the dietary recommendations they are associated with may offer further useful insights and could be studied further.

## Conclusion

We used data mining techniques to explore the number of foods that consumption information was required on to accurately predict achievement, or not, of five key dietary recommendations. Information on consumption of eleven to thirty-two foods (plus age and sex) was sufficient to identify with 72–83 % accuracy whether individuals achieved individual dietary recommendations. In total, information on 113 foods was required to predict achievement of all five recommendations studied. This method could be used to develop a new dietary assessment questionnaire.

## Supplementary material

For supplementary material accompanying this paper visit http://dx.doi.org/10.1017/S1368980016000185. click here to view supplementary material
