# Supplementary material for: Identifying small groups of foods that can predict achievement of key dietary recommendations: data mining of the UK National Diet and Nutrition Survey, 2008–12
Source: Public Health Nutr. 2016 Feb 16;19(9):1543–51. doi: 10.1017/S1368980016000185 (PMC4873899; doi:10.1017/S1368980016000185)
Supplement: Supplementary file 1 [file S1368980016000185sup001.docx]

**SUPPLEMENTARY MATERIAL: CLASS BALANCING**

Classes in the NDNS dataset were not balanced: one class had up to 4.2 times as many instances as another in the case of meeting guidelines for saturated fat, and the most balanced distribution still had 1.5 times as many of one instance than another in the case of meeting guidelines for salt. As noted by Poolsawad and colleagues, "medical data commonly has an imbalanced class distribution [...] On such data, learning classification methods generally perform poorly because the classifier often learns better the majority class".^(1)^ Indeed, in an unbalanced distribution, classifiers can simply label all individuals as belonging to one class and still achieve a low error rate when that class is sufficiently prevalent. This was expected and observed in our case (Supplemental Table 1).

**Supplemental Table 1. Prevalence of meeting/not meeting the guidelines for each of the 5 guidelines.**

|  | Fruits & Veg. | Free sugars | Salt | Fat | Saturated Fat |
| --- | --- | --- | --- | --- | --- |
| Prevalence: YES | 656* | 1472 | 2524 | 1045 | 795 |
| Prevalence: NO | 2311* | 2684 | 1632 | 3111 | 3361 |
| Imbalance** | 3.5* | 1.8 | 1.5 | 3.0 | 4.2 |
| Overall accuracy*** | 81.86 | 74.37 | 74.03 | 74.13 | 79.52 |
| Accuracy on YES | 44.4 | 55.4 | 82.8 | 0.06 | 18.5 |
| Accuracy on NO | 92.5 | 84.8 | 60.4 | 96.8 | 94.0 |

*Guidelines for fruits and vegetables are only available for children aged 11 years and older

**Imbalance is the ratio between the majority and the minority classes

***The classifier was a J4.8 decision tree with error pruning and a minimum of 5 instances per leaf.

This problem arises in part because classifiers assume that the training data is balanced and that errors have the same cost.^(1; 2)^ Thus, getting all minority classes wrong but the one majority class right is 'as good' from the viewpoint of a classifier as recognizing most minority classes but less of the majority class. However, from a clinical decision-making viewpoint, the objective is to find patterns in the data such that we can understand why individuals end up in a certain class, rather than blindly assign them label without finding mechanisms. Note that other issues than imbalance can affect classification errors, such as class overlaps; these issues are beyond the scope of this manuscript and we refer the reader to (Japkowicz 2003) for a more in-depth discussion.^(3)^

There are mostly three ways to address the problem of class imbalances: eliminating cases from the majority class (under-sampling), creating new cases for the minority class (over-sampling), or biasing the classifier's algorithm (e.g., using non-uniform error costs depending on class imbalance). These techniques were reviewed by Mollineda et al., 2007.^(4)^ Here, we use the J48 classifier from WEKA, as it is one of the most commonly used classifiers.^(2)^ Thus, rather than modifying the algorithm, we used sampling techniques to address the problem of class imbalances. A comparison of sampling methods Batista et al. (2005) concluded that "over-sampling methods in general, and SMOTE-based methods in particular" were very efficient.^(5)^ Thus, we used the Synthetic Minority Over-sampling TEchnique (SMOTE), which creates new cases for the minority class by interpolating between existing cases that lie together.^(6)^ We used SMOTE 1.0.3 for Weka 3.7.12.

**References**

1. Poolsawad N, Kambhampati C, Cleland J (2014) Balancing class for performance of classification with a clinical dataset. *Proceedings of the World Congress on Engineering (WCE)*.

2. Rahman M, Davis D (2013) Cluster based under-sampling for unbalanced cardiovascular data. *Proceedings of the World Congress on Engineering (WCE)*.

3. Japkowicz N (2003) Class imbalances: are we focusing on the right issue? *ICML'2003 Workshop on Learning from Imbalanced Data Sets*.

4. Mollineda R, Alejo R, Sotoca J (2007) The class imbalance problem in pattern classification and learning*. *II Congreso Español de Informática (CEDI 2007)*.

5. Batista GAPA, Prati R, Monard M (2005) Balancing Strategies and Class Overlapping. In *Advances in Intelligent Data Analysis VI*, vol. 3646, pp. 24-35 [AF Famili, J Kok, J Peña, A Siebes and A Feelders, editors]: Springer Berlin Heidelberg.

6. Chawla N, Bowyer K, Hall L *et al.* (2002) SMOTE: Synthetic Minority Over-sampling Technique. *Journal of Artificial Intelligence Research* **16**, 321-357.
